# Neonatal Mortality Rate and Its Determinants: A Community–Based Panel Study in Ethiopia

**DOI:** 10.3389/fped.2022.875652

**Published:** 2022-05-23

**Authors:** Kasiye Shiferaw, Bezatu Mengistie, Tesfaye Gobena, Merga Dheresa, Assefa Seme

**Affiliations:** ^1^School of Nursing and Midwifery, College of Health and Medical Sciences, Haramaya University, Harar, Ethiopia; ^2^School of Public Health, St. Paul's Hospital Millennium Medical College, Addis Ababa, Ethiopia; ^3^Department of Environmental Health Science, College of Health and Medical Sciences, Haramaya University, Harar, Ethiopia; ^4^School of Public Health, Addis Ababa University, Addis Ababa, Ethiopia

**Keywords:** survival, prevalence, neonate, predictors, Ethiopia

## Abstract

**Background:**

The Sustainable Development Goals specifically target a reduction in neonatal mortality rates. However, the highest neonatal mortality rates occur in sub-Saharan Africa, including Ethiopia. Although several factors contributing to these high rates have been explored, there continues to be a general dearth of studies and inconsistencies of factors to understand the problem. Therefore, this study aimed to identify the prevalence and factors associated with neonatal mortality in Ethiopia.

**Methods:**

A panel study was conducted among 2,855 pregnant or recently postpartum women selected using the multistage cluster sampling technique from October 2019 to September 2020. Data were collected by experienced and trained female resident enumerators and coded, cleaned, and analyzed using STATA version 16.1 software. We used the Kaplan–Meier survival curve to show the pattern of neonatal deaths during the first 28 days of life. Frequencies and rates were reported along with the percentages and using a 95% confidence interval, respectively. The Cox proportional hazard regression model was used to explore the association of explanatory and outcome variables. Finally, an adjusted hazard ratio with a 95% confidence interval was used to report the results, with a *p* < 0.05 to declare statistical significance.

**Results:**

The neonatal mortality rate was 26.84 (95% CI: 19.43, 36.96) per 1,000 live births. Neonates born to rural resident mothers (AHR = 2.18, 95% CI: 1.05, 4.54), mothers of advanced age (AHR = 2.49, 95% CI: 1.19, 5.21), and primipara mothers (AHR = 3.16, 95% CI: 1.52, 6.60) had a higher hazard of neonatal mortality. However, neonates born to women who attended technical and vocational level education (AHR = 0.08, 95% CI: 0.01, 0.62) had a lower hazard of neonatal mortality.

**Conclusions:**

The neonatal mortality rate in Ethiopia is high, with increased risk among specific subsets of the population. The findings highlight that neonatal survival can be improved through tailored interventions for rural residents, emerging regions, and primipara women by improving female education and avoiding pregnancy at an advanced maternal age to achieve Sustainable Development Goal target 3.2.

## Background

The Sustainable Development Goal (SDG) 3.2 targets to reduce neonatal mortality (NM) to 12 deaths per 1,000 live births or lower by 2030; however, accelerated progress is needed by countries to reach this goal (1, 2). Neonatal mortality is death among neonates during the first 28 days of life after a live birth (3). The first month is critical for newborn survival (4). Since neonatal mortality contributes to the majority of under-5 mortality, it is a relevant indicator of children's wellbeing and health (1). The global neonatal mortality decreased from 5 to 2.5 million from 1990 to 2017; however, the annual NM rate fluctuates. The NM rate was 27 per 1,000 live births in sub-Saharan Africa (SSA) (4) and 30 per 1,000 live births in Ethiopia, with slight improvement (5, 6).

Sociodemographic factors, reproductive health, perinatal care, and child-feeding practice contribute to the high NM rate (4, 7, 8). Specifically, the women's level of education (9–11), place of residence (9, 12, 13), parity (14, 15), delivery place (10), newborn age (16), women's age (16–18), newborn sex (11, 19), gestational age (16), maternal or fetal complications (20, 21), delivery mode (20–22), low birth weight (21, 23, 24), low Apgar score (14, 23), congenital abnormalities (21, 23), late initiation of breastfeeding (16, 19, 20), non-exclusive breastfeeding (16), and inadequate antenatal care (ANC) visits (16, 20, 24) were associated with neonatal mortality across low- and middle-income countries (LMICs).

The pooled NM prevalence in Ethiopia varied from 6.8 (25) to 16.3% (7), which is unacceptably high. Therefore, a continuum of care is recommended to improve neonatal survival (26). Current studies in Ethiopia are limited to specific geographical locations (27, 28) and are primarily facility-based (12, 16, 17, 29–32); hence, it is challenging to estimate NM's national burden and predictors. Additionally, current pieces of evidence on incidence and predictors of NM were limited and at the national level, based on the demographic health survey (DHS), which is subject to recall bias since it depends on the women's recall of the past 5 years (13, 19, 33, 34). Hence, there is a general dearth of studies and inconsistencies in understanding the problems contributing to NM. Therefore, this study aimed to identify the prevalence and factors associated with neonatal mortality in Ethiopia.

## Materials and Methods

### Study Design, Setting, and Period

A panel design was conducted in six regions of Ethiopia, Tigray, Afar, Amhara, Oromia, South Nation Nationalities and People (SNNP), and Addis Ababa city administration. These regions represent ~90% of the country's total population (35). The study was conducted from October 2019 to September 2020.

### Sample Size and Sampling Population

The final sample size in this panel study was 2,855, which was nested within 217 enumeration areas. First, the roster of all households was created in the community to identify eligible females for the panel study during the census. Then, eligible women were identified and enrolled in the panel study using a screening form. The pregnant or recently postpartum (<8 weeks postpartum with a live birth) women living in the panel regions were eligible. In addition, if a woman was staying at her parents' home during the census and screening, she qualified (even though this was not her permanent residence).

### Variables

Neonatal mortality was the outcome variable. The women who had a live birth but lost their neonate within the first 28 days postpartum were coded as “1”, otherwise, it was considered “0”. The explanatory variables included women's age (15–19, 20–24, 25–29, 30–34, ≥35 years), place of residence (urban or rural), wealth quintile (lowest, lower, middle, higher, or highest), level of education (never attended, primary, secondary, technical, and vocational or higher), dwelling region (Tigray, Afar, Amhara, Oromia, SNNP, Addis Ababa), religion (Orthodox, Muslim, Protestant, others), marital status (married or others), parity (primipara, multipara), ever been pregnant (yes or no), ANC attendance (yes or no) and sex of neonate (male or female).

Advanced maternal age was defined as women aged 35 and above (36). We calculated the wealth quintile from household assets and housing conditions. Hence, households were given a score based on the number and types of goods and housing characteristics. The principal component analysis derived the scores. The scores were divided into five equal categories (5). Urban was defined as a locality with 2,000 or more inhabitants. However, it also included district capitals, localities with urban dweller associations, and localities primarily engaged in non-agrarian activities with 1,000 or more inhabitants (37). The women's marital status was categorized based on her response to the question ‘are you currently married or living together with a man as if married?' The woman's marital status was labeled as “married” if her response was currently married or living with a man, on the other hand, if she responded that she was divorced, separated, widowed, or never married, it was categorized as “others.”

### Data Collection

The tools were adapted from the demographic health surveys, and previous Performance Monitoring for Action (PMA) Ethiopia tools and developed by reviewing the literature. The tool is available on the PMA website (https://www.pmadata.org/data/survey-methodology). Experienced, trained, female resident enumerators collected the data using an interviewer-administered questionnaire. First, a baseline assessment was conducted following the enrollment of the participants. Then, for baseline data collection, wave two data collection was conducted from 5 to 8 weeks postpartum, both for pregnant or recently postpartum women.

### Data Quality Assurance

A validated questionnaire adapted from the demographic health survey tool, previous PMA Ethiopia tool, and reviewing literature were pretested in the Oromia zone; results were excluded in the actual data analysis. Diploma data collectors and supervisors were recruited. The data collectors had varying years of prior experience among themselves. Many participated in the DHS; additionally, some data collectors had worked with the longstanding cross-sectional PMA in Ethiopia (ongoing since 2014) and a similar panel study in SNNP in 2016. Female resident enumerators and supervisors received comprehensive training on data collection tools and procedures for 2 weeks. The consented study participants were repeatedly contacted during round two data collection (i.e., three visits as needed) and provided with an incentive mobile card to minimize loss of follow-up. The data were downloaded from the aggregate server daily and cleaned using STATA version 16.1 software by the data management team.

### Data Processing and Analysis

Data were downloaded to STATA version 16.1 software for analysis. Frequency tables and Kaplan–Meier curves were used during descriptive analysis. Unequal clusters and women's probability selection and non-response bias were compensated by sample weights (women and household). The sample weight was the output of inverse household and enumeration areas selection probability, female, and household response rate in this study.

The outcome was a dichotomous variable coded as one when NM occurred within 28 days of delivery after live birth. Otherwise, it was coded as zero. The time to death was calculated by subtracting the birth date from the date of death. The neonatal mortality rate was calculated as neonatal deaths per 1,000 live births. The neonatal mortality rate was described each year with a 95% confidence interval (CI). Kaplan–Meier survival curve was used to show the neonatal death patterns, and a log-rank test was used to compare the survival curves among the independent variables. The variance inflation factor (vif) indicated no multi-collinearity of explanatory variables (i.e., vif = 1.31). Most of the missing values were participants moving outside the study area, lost forms, and failure to locate the women within 8 weeks postpartum after repeated visits. The missing data, loss of follow-up, and causes were evaluated and not felt to threaten the study's validity. The Cox proportional hazard regression model was employed to identify the association of explanatory variables with NM by consecutive backward elimination. The test of proportional hazard assumption was checked based on the Schoenfeld residuals test (i.e., *p*-value = 0.24). Adjusted hazard ratio (AHR) with a 95% CI was used to report the results. A *p* < 0.05 was considered to declare statistical significance.

## Results

**Figure 1:**
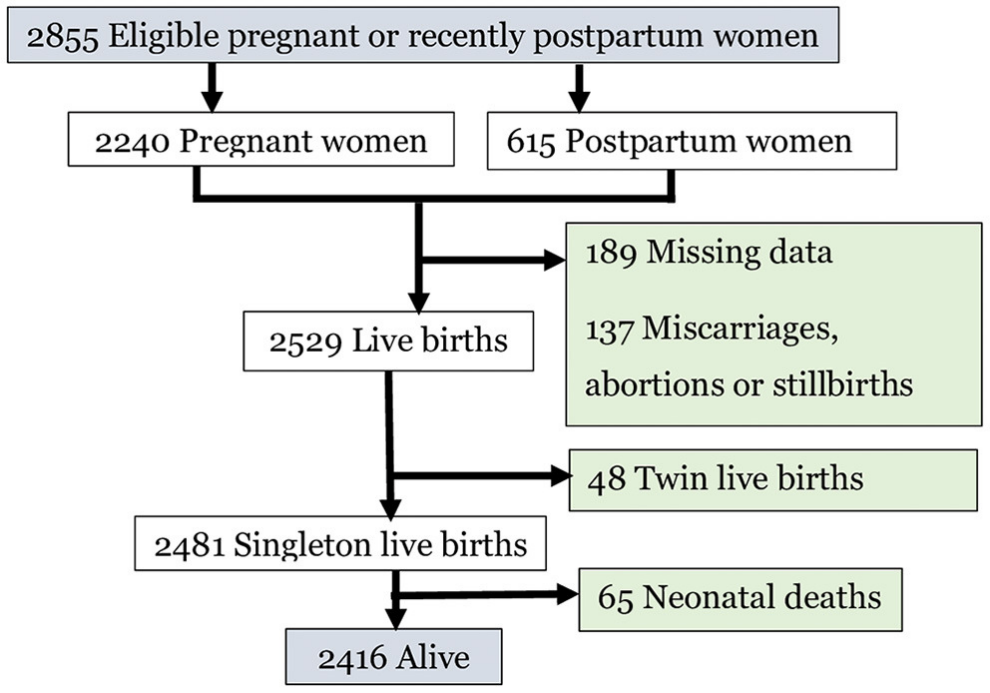
Pregnant or recently postpartum women enrolled, pregnancy outcomes and retained participants for analysis, 2020.

Among the 2,855 women enrolled in the panel study, 2,240 were currently pregnant, 338 recently postpartum, and 277 were 5–9 weeks postpartum. Finally, 2,481 singleton live birth neonates born to these women were considered in this analysis (Figure 1).

### Characteristics of Participants

The mean age of the participants was 27.11 (± 6.11) years. A total of eight hundred twelve (30.41%) women were between the ages of 25 and 29, 2,077 (77.1%) were rural residents, and 1,100 (41.2%) had no formal education. Further, 1,560 (82.70%) participants were multiparas, and 498 (20.06%) were from the middle wealth quintile (Table 1).

**Table 1:** Frequency and percentage of study participants' characteristics in Ethiopia 2020 (*n* = 2,481).

**Variable**	**Un-weighted frequency (%)**	**Weighted frequency (%)**
**Maternal age**
15–19	250 (9.36)	280 (10.50)
20–24	688 (25.77)	659 (24.69)
25–29	851 (31.87)	812 (30.41)
30–34	483 (18.09)	483 (18.09)
≥35	398 (14.91)	435 (16.31)
**Residence**
Urban	1,073 (39.8)	615 (22.9)
Rural	1,621 (60.2)	2,077 (77.1)
**Region**		
Tigray	420 (16.93)	172 (6.93)
Afar	198 (7.98)	46 (1.83)
Amhara	436 (17.57)	521 (21.01)
Oromia	622 (25.07)	1,099 (44.29)
SNNP	563 (22.69)	551 (22.22)
Addis Ababa	242 (9.75)	92 (3.72)
**Wealth quintile**		
Lowest	442 (17.82)	516 (20.81)
Lower	373 (15.03)	496 (20.00)
Middle	385 (15.52)	498 (20.06)
Higher	460 (18.54)	490 (19.76)
Highest	821 (33.09)	481 (19.37)
**Religion**		
Orthodox	1,158 (46.67)	975 (39.29)
Muslim	742 (29.91)	846 (34.09)
Protestant	550 (22.17)	611 (24.61)
Others^a^	31 (1.25)	50 (2.01)
**Level of education**
Never attended	946 (38.13)	1,043 (42.06)
Primary	895 (36.07)	982 (39.59)
Secondary	372 (14.99)	279 (11.24)
Technical or vocational	105 (4.23)	79 (3.17)
Higher	163 (6.57)	98 (3.94)
**Marital status**
Others^b^	175 (6.55)	135 (5.04)
Married	2,495 (93.45)	2,534 (94.96)
**Parity**
Primipara	480 (19.35)	429 (17.30)
Multipara	2,001 (80.65)	1,560 (82.70)
**Ever been pregnant**
No	146 (5.47)	127 (4.74)
Yes	2,524 (94.53)	2,542 (95.26)
**Antenatal care visit**		
No	781 (31.48)	787 (31.72)
Yes	1,700 (68.52)	1,694 (68.28)
**Sex of neonate**		
Male	1,238 (50.65)	1,261 (51.60)
Female	1,206 (49.35)	1,183 (48.40)

*a, Catholic or Traditional or Wakefata; b, widowed or divorced or separated or never married, Weighted, calculated considering weighting factor; Un-weighted, calculated without considering weighting factor*.

### Neonatal Mortality Rate

**Figure 2:**
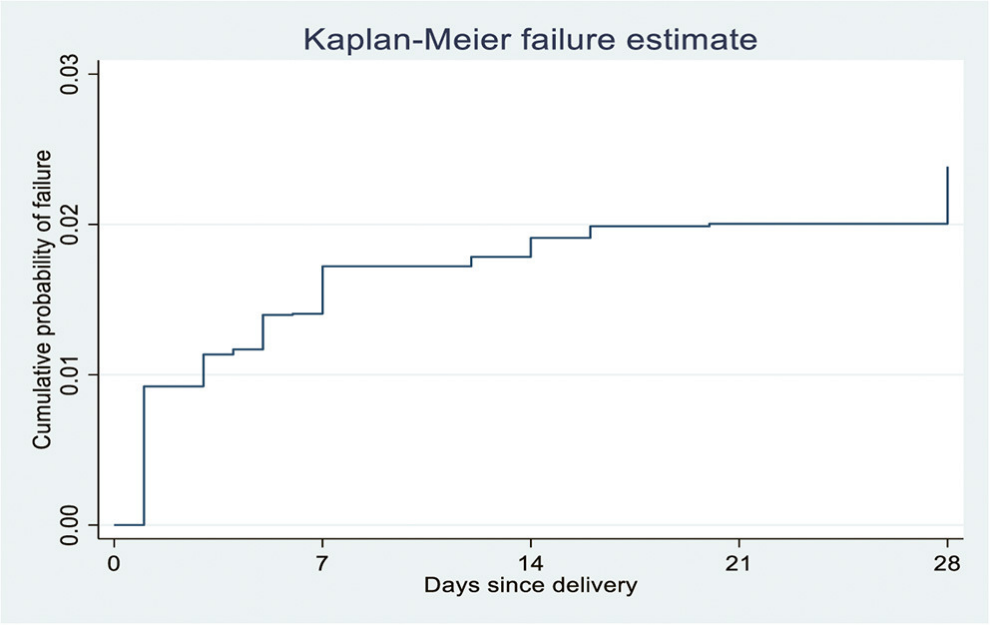
Kaplan-Meier failure estimates of neonatal mortality per day since delivery in Ethiopia, 2020 (*n* = 2481).

The study revealed that the NM rate was 26.84 (95% CI: 19.43, 36.96) per 1,000 live births. During the study period, 2.68% (95% CI: 1.94–3.69%) (*n* = 65) of the neonates died among 2,481 singleton live births. A total of thirty-two (54%) and 47 (80%) neonatal deaths occurred within the first 2 and 7 days of life, respectively (Figure 2).

The log-rank test indicated significant variation of survival pattern to neonatal mortality over the place of residence (X^2^ for log-rank test =11.54, *p* < 0.01), wealth quintile (X^2^ for log-rank test =11.93, *p* = 0.01), maternal age (X^2^ for log-rank test = 14.41, *p* < 0.01), previous pregnancy (X^2^ for log-rank test = 5.80, *p* = 0.01), parity (X^2^ for log-rank test = 5.60, *p* = 0.01), and attending at least one ANC visit (X^2^ for log-rank test = 4.47, *p* = 0.03; Table 2).

**Table 2:** Result of log-rank test for neonates born to Ethiopian women, October 2019 to September 2020 (*n* = 2,481).

**Variable**	**Log-rank**	***P*-value**
Residence	11.54	<0.01
Region	7.35	0.19
Wealth quintile	11.93	0.01
Religion	6.59	0.08
Maternal age	14.41	<0.01
Educational level	2.95	0.39
Ever been pregnant	5.80	0.01
Parity	5.60	0.01
Antenatal care visit	4.47	0.03
Sex of neonate	2.91	0.08

### Factors Associated With Neonatal Survival

The multivariable analysis indicated that the place of residence, region of dwelling, education level, maternal age, and parity have a significant association with neonatal survival. The neonates born to women of advanced age have two times higher hazard of NM compared to neonates born to women aged 15–19 years (AHR = 2.49, 95% CI: 1.19, 5.21). The hazard of NM was two times higher among neonates born to rural resident women than their urban resident counterparts (AHR = 2.18, 95% CI: 1.05, 4.54). The risk of neonatal mortality was two times higher among neonates born to women living in the Afar region than those in the Tigray region (AHR = 2.42, 95% CI: 1.04, 5.62). Similarly, neonates born to primipara mothers had three times higher hazard of NM compared to neonates born to multipara counterparts (AHR = 3.16, 95% CI: 1.52, 6.60).

The hazard of NM among educated women (to the level of technical and vocational training) was 92% lower than those who never attended formal education (AHR = 0.08, 95% CI: 0.01, 0.62; Table 3).

**Table 3:** Multivariable Cox proportional hazard regression analysis of predictors of neonatal mortality in Ethiopia, October 2019 to September 2020 (*n* = 2,481).

**Variable**	**Neonatal survival**		**CHR**	**AHR**
	**Censored (%)**	**Died (%)**	**(95% CI)**	**(95% CI)**
**Residence**
Urban	535 (98.73)	7 (1.27)	1.00	1.00
Rural	1,879 (96.92)	60 (3.08)	2.14 (1.04, 4.39)*	2.18 (1.05, 4.54)*
**Region**				
Tigray	167 (97.36)	5 (2.64)	1.00	1.00
Afar	43 (95.31)	3 (4.69)	1.61 (0.60, 4.33)	2.42 (1.04, 5.62)*
Amhara	508 (97.47)	13 (2.53)	0.75 (0.28, 2.05)	0.71 (0.26, 1.95)
Oromia	1,067 (97.13)	32 (2.87)	1.04 (0.44, 2.44)	1.06 (0.46, 2.46)
SNNP	537 (97.47)	14 (2.53)	0.81 (0.32, 2.00)	0.74 (0.29, 1.87)
Addis	91 (98.69)	2 (1.31)	0.49 (0.13, 1.85)	0.82 (0.18, 3.59)
**Woman's age**
15–19	240 (96.29)	9 (3.71)	1.00	1.00
20–24	586 (98.19)	11 (1.81)	0.51 (0.16, 1.62)	0.93 (0.32, 2.72)
25–29	746 (97.88)	16 (2.12)	0.58 (0.19, 1.77)	1.82 (0.61, 5.38)
30–34	443 (97.28)	12 (2.72)	0.67 (0.20, 2.23)	2.54 (0.68, 4.42)
≥35	400 (95.67)	18 (4.31)	1.23 (0.42, 3.56)	2.49 (1.19, 5.21)*
**Level of education**
NAS	1,015 (97.30)	28 (2.70)	0.98 (0.27, 3.51)	0.65 (0.14, 2.89)
Primary	951 (96.85)	30 (3.15)	1.25 (0.35, 4.47)	1.13 (0.28, 4.58)
Secondary	274 (98.19)	5 (1.81)	0.81 (0.19, 3.41)	0.93 (0.18, 4.67)
TeVT	79 (99.81)	2 (1.90)	0.08 (0.01, 0.81)*	0.08 (0.01, 0.62)*
Higher	96 (97.69)	3 (2.31)	1.00	1.00
**Wealth quintile**				
Lowest	507 (98.21)	9 (1.79)	0.98 (0.40, 2.40)	0.64 (0.23, 1.79)
Lower	474 (95.60)	22 (4.40)	2.32 (0.99, 5.45)	1.50 (0.56, 4.05)
Middle	477 (95.80)	21 (4.20)	2.30 (0.89, 5.39)	1.45 (0.52, 4.00)
Higher	483 (98.60)	7 (1.40)	0.83 (0.30, 2.32)	0.39 (0.15, 0.99)*
Highest	473 (98.38)	8 (1.62)	1.00	1.00
**Ever been pregnant**
No	103 (92.80)	8 (7.20)	2.55 (1.15, 5.61)*	2.55 (0.86, 7.52)
Yes	2,311 (97.53)	59 (2.47)	1.00	1.00
**Birth events**
Primipara	411 (95.66)	19 (4.34)	2.02 (1.04, 3.89)*	3.16 (1.52, 6.60)*
Multipara	2004 (97.96)	48 (2.04)	1.00	1.00

CHR, Crude Hazard Ratio; AHR, Adjusted Hazard Ratio; CI, Confidence Interval; NAS, Never Attended School; TeVT, Technical and Vocational Training; Censored – Survived neonates, *Significant at P-value < 0.05 levels; 1.00 – Reference.

## Discussion

This study identified the NM rate and factors associated with neonates born to Ethiopian women; the prevalence in Ethiopia was high. Additionally, the factors significantly associated with neonatal mortality were advanced maternal age, rural residence, and primiparity.

The NM rate among neonates born to Ethiopian women was 26.84 per 1,000 live births. Similarly, studies across Ethiopia revealed NM rates of 31.6 (38), 27 (17), and 20.7 (19) per 1,000 live births. The mini DHS 2019 (6), DHS 2016 (5), and analysis based on the DHS (34) also revealed NM rates of 30, 29, and 29 per 1,000 live births, respectively, in Ethiopia. Further, a study in the Somali region of Ethiopia revealed that the NM rate was 57 per 1,000 live births (30). Several factors like prematurity, congenital abnormalities, maternal malnutrition, perinatal asphyxia, and sepsis may be contributary to the high rate (12).

Further, low-health service coverage (i.e., continuum of care), including inaccessible quality maternal and child care, skilled birth attendants, postnatal care, and sociodemographic factors, might contribute to the variations (5). Studies have shown that the NM rate in Ethiopia has been stagnating for a decade. However, the Sustainable Development Goal 3.2 targets the NM rate to reduce the deaths to at least as low as 12 per 1,000 live births by 2030 (39), and the Ethiopian Health Sector Transformation Plan II plans to decrease the NM rate to 21 per 1,000 live births by 2025 (40). Hence, our study alerts policy designers and program implementers that tailored interventions are crucial to reducing the NM to achieve these targets.

The study indicates neonates born to rural residents have an increased hazard of NM compared to their urban counterparts. Similarly, previous studies conducted in Ethiopia (12, 13) found neonates from rural areas have increased chances of death early in life compared to those living in urban areas. Further, women who are uneducated and residing in rural areas are less likely to attend and receive all ANC services available (41), resulting in less or non-use of the continuum of care among rural women, and thus the high risk for NM (9, 12, 42). This leaves significant room for policy development, policies to support neonates born to women in rural areas.

In this study, neonates born to women of advanced age have an increased hazard of NM compared to those aged 15–19 years. Similarly, advanced maternal age has increased the odds of NM in Uganda (15) and Ethiopia (17). A study in Afghanistan also showed advanced maternal age was associated with an increased NM (18). In addition, advanced maternal age is associated with low birth weight (43, 44), preterm (44–47), and other pregnancy complications (47–49), which are associated with NM (12, 50, 51). Further, a study indicated advanced maternal age was associated with unexplained neonatal death (52). This finding highlights the significant role of reducing pregnancy at an advanced maternal age as a strategy to improve neonatal survival.

Neonates born to educated women, who attended vocational and technical training, had a decreased hazard of NM. Similarly, those neonates born to women whose mothers could not read and write had an increased hazard of NM (17). A study in Afghanistan also indicated neonates born to women with higher educational levels had decreased NM (18). This might be due to educated women being more likely to seek healthcare, be autonomous, and be aware of neonatal danger signs. This highlights the fact that female education plays a crucial role to reduce the hazard of NM in Ethiopia.

In this study, neonates born to primipara women have a high hazard of NM compared to their multipara counterparts. A study revealed that neonates born to multipara women had 42% lesser odds of dying compared to their primipara counterparts (32). Similarly, the odds of losing newborns among neonates born to women whose parity was 2–4 and five or more children were lesser than a primipara (28). Furthermore, multiparity reduced the NM by 30% in Ghana and South Africa (53, 54). The odds of delivering a low birth weight newborn were low among multigravida women (55). Multipara women practiced optimal breastfeeding more often than primipara (56, 57), contributing to a lower NM (58). Timely initiation and exclusive breastfeeding may contribute to the prevention of diarrhea-related NM rates (59). The finding highlights the fact that tailored interventions for primipara women are crucial to improving neonatal survival.

### Strength and Limitations of the Study

This study utilized nationally representative data and a community-based panel design. Sample weights (household and female) were constructed to minimize selection bias, i.e., compensate for unequal probability selection of clusters and women and non-response bias, thereby, ensuring generalizability. However, recall bias cannot be ruled out since the women have to remember some of the events retrospectively. Misclassification of some stillbirths as early neonatal deaths and loss of follow-up due to failure to locate, movement out of EAs, and death of the women may have occurred in this study. The loss of follow-up was declared after repeated visits to the participants (i.e., maximum of three times), as a result, variables with extensive missing data were not considered during analysis. This panel study did not assess factors such as congenital anomalies, birth trauma, autopsy, interpregnancy interval, and history of neonatal deaths that may predict neonatal mortality. Additionally, further research should assess the relationship between the timeliness, frequency, or contents of ANC visits and NM in the future.

## Conclusions

The NM rate among neonates born to Ethiopian women was unacceptably high. The findings highlight that neonatal survival can be improved through tailored interventions for rural residents, emerging regions, and primipara women, promoting female education and avoiding pregnancy at an advanced maternal age.

## Data Availability Statement

The datasets presented in this article are not readily available because the PMA datasets policy does not allow to publish results that communities or individual can be identified or datasets are anonymized before it is made publicly available. It is forbidden to make an effort to identify individual, household, or enumeration areas in the survey, and use the data for marketing and commercial ventures. Access to datasets is granted by PMA Ethiopia upon reviewing the submitted request via www.pmadata.org. Further enquires can be directed to the corresponding author.

## Ethics Statement

Ethical approval was received from Addis Ababa University, College of Health Sciences (AAU/CHS) (Ref: AAUMF 01-008), and the Johns Hopkins University Bloomberg School of Public Health (JHSPH) Institutional Review Board (FWA00000287) by PMA Ethiopia (35). The PMA datasets policy does not allow to publish results that communities or individual can be identified or datasets are anonymized before it is made publicly available. It is forbidden to make an effort to identify individual, household, or enumeration areas in the survey, and use the data for marketing and commercial ventures. Access to datasets is granted by PMA Ethiopia upon reviewing the submitted request. Informed, voluntary consent was obtained from all participants. Thus, all methods were carried out following relevant guidelines and regulations.

## Author Contributions

KS conceived, designed, analyzed, and prepared a draft of the manuscript. All authors participated in study design, acquisition of data, analysis and interpretation, critical review of the document, and revision of the manuscript. All authors contributed to the article and approved the submitted version.

## Conflict of Interest

The authors declare that the research was conducted in the absence of any commercial or financial relationships that could be construed as a potential conflict of interest.

## Publisher's Note

All claims expressed in this article are solely those of the authors and do not necessarily represent those of their affiliated organizations, or those of the publisher, the editors and the reviewers. Any product that may be evaluated in this article, or claim that may be made by its manufacturer, is not guaranteed or endorsed by the publisher.

## Abbreviations

AHR: Adjusted hazard ratio
ANC: antenatal care
CHR: Crude hazard ratio
EDHS: Ethiopian demographic health survey
HCP: health care provider
HEWs: health extension workers
NM: neonatal mortality
NMR: neonatal mortality rate
PNC: postnatal care
SDG: Sustainable Development Goal
TeVT: Technical and vocational training
WHO: world health organization.
